# Cohesin mediates Esco2-dependent transcriptional regulation in a zebrafish regenerating fin model of Roberts Syndrome

**DOI:** 10.1242/bio.026013

**Published:** 2017-10-30

**Authors:** Rajeswari Banerji, Robert V. Skibbens, M. Kathryn Iovine

**Affiliations:** Department of Biological Science, Lehigh University, Bethlehem, Pennsylvania 18015, USA

**Keywords:** Roberts Syndrome, Cornelia de Lange syndrome, Cohesin, *esco2*, *smc3*, *cx43*, Zebrafish, Regeneration, Transcription

## Abstract

Robert syndrome (RBS) and Cornelia de Lange syndrome (CdLS) are human developmental disorders characterized by craniofacial deformities, limb malformation and mental retardation. These birth defects are collectively termed cohesinopathies as both arise from mutations in cohesion genes. CdLS arises due to autosomal dominant mutations or haploinsufficiencies in cohesin subunits (*SMC1A*, *SMC3* and *RAD21*) or cohesin auxiliary factors (*NIPBL* and *HDAC8*) that result in transcriptional dysregulation of developmental programs. RBS arises due to autosomal recessive mutations in cohesin auxiliary factor *ESCO2*, the gene that encodes an N-acetyltransferase which targets the SMC3 subunit of the cohesin complex. The mechanism that underlies RBS, however, remains unknown. A popular model states that RBS arises due to mitotic failure and loss of progenitor stem cells through apoptosis. Previous findings in the zebrafish regenerating fin, however, suggest that Esco2*-*knockdown results in transcription dysregulation, independent of apoptosis, similar to that observed in CdLS patients. Previously, we used the clinically relevant *CX43* to demonstrate a transcriptional role for Esco2. *CX43* is a gap junction gene conserved among all vertebrates that is required for direct cell-cell communication between adjacent cells such that *cx43* mutations result in oculodentodigital dysplasia. Here, we show that morpholino-mediated knockdown of *smc3* reduces *cx43* expression and perturbs zebrafish bone and tissue regeneration similar to those previously reported for *esco2* knockdown. Also similar to Esco2-dependent phenotypes, Smc3-dependent bone and tissue regeneration defects are rescued by transgenic Cx43 overexpression, suggesting that Smc3 and Esco2 cooperatively act to regulate cx43 transcription. In support of this model, chromatin immunoprecipitation assays reveal that Smc3 binds to a discrete region of the *cx43* promoter, suggesting that Esco2 exerts transcriptional regulation of *cx43* through modification of Smc3 bound to the *cx43* promoter. These findings have the potential to unify RBS and CdLS as transcription-based mechanisms.

## Abstract (precis)

**Summary:** This study reveals an underlying mechanism of RBS in which *ESCO2* mutation results in cohesin-dependent dysregulation of transcriptional programs that include the clinically relevant signaling molecule *CX43*. This article has an associated First Person interview with the first author of the paper as part of the supplementary information.

## INTRODUCTION

Roberts syndrome (RBS) is a multi-spectrum developmental disorder characterized by severe skeletal deformities resulting in craniofacial abnormalities, long-bone growth defects and mental retardation (Van den Berg and Francke, 1993; Vega et al., 2005). Infants born with severe forms of RBS are often still-born and even modest penetrance of RBS phenotypes lead to significantly decreased life expectancy (Schüle et al., 2005). Cornelia de Lange Syndrome (CdLS) patients exhibit phenotypes similar to RBS patients, including severe long-bone growth defects, missing digits, craniofacial abnormalities, organ defects and severe mental retardation (Tonkin et al., 2004; Krantz et al., 2004; Gillis et al., 2004; Musio et al., 2006). Collectively, RBS and CdLS are termed cohesinopathies as they arise due to mutations in genes predominantly identified for their role in sister chromatid tethering reactions (termed cohesion) (Vega et al., 2005; Schüle et al., 2005; Gordillo et al., 2008; Krantz et al., 2004; Musio et al., 2006; Tonkin et al., 2004; Deardorff et al., 2007, 2012a,b). Cohesins are composed of two structural maintenance of chromosome (SMC) subunits, SMC1A and SMC3, and several non-SMC subunits that include RAD21 (Mcd1/Scc1), SA1, 2 (stromal antigen/Scc3/Irr1) and PDS5. At least a subset of cohesin subunits form rings that appear to topologically entrap individual DNA segments (Guacci et al., 1997; Michaelis et al., 1997; Toth et al., 1999; Hartman et al., 2000; Panizza et al., 2000; Haering et al., 2002; Gruber et al., 2003; Arumugam et al., 2003; Tong and Skibbens, 2014; Eng et al., 2015; Stigler et al., 2016).

RBS is an autosomal recessive disease that arises due to loss of function mutations in the *ESCO2* gene that encodes an N-acetyltransferase (Ivanov et al., 2002; Bellows et al., 2003; Hou and Zou, 2005; Vega et al., 2005). *ESCO2*/*EFO2* (and *ESCO1*/*EFO1* paralog) are the human orthologues of the *ECO1*/*CTF7* first identified in budding yeast (Skibbens et al., 1999; Toth et al., 1999; Bellows et al., 2003; Hou and Zou, 2005). All ESCO/EFO family N-acetyltransferases modify the SMC3 cohesin subunit (Zhang et al., 2008; Unal et al., 2008; Rolef Ben-Shahar et al., 2008). ESCO2 plays an essential role in sister chromatid cohesion during S phase and ensures proper chromosome segregation during mitosis. In contrast, CdLS arises due to autosomal dominant mutations in cohesin subunits (*SMC1A*, *SMC3* and *RAD21*) and cohesin auxiliary factors (*NIPBL* and *HDAC8*) (Krantz et al., 2004; Tonkin et al., 2004; Schüle et al., 2005; Musio et al., 2006; Deardorff et al., 2007, 2012a,b; Gordillo et al., 2008; Yuan et al., 2015). NIPBL/Scc2 and MAU2/Scc4 heterodimer complex are required for cohesin ring opening/closing reactions that load cohesins onto DNA (Ciosk et al., 2000; Arumugam et al., 2003; Watrin et al., 2006; Bernard et al., 2006).

Extensive research provides fascinating evidence that cohesin functions beyond sister chromatid cohesion (*trans*-tethering that brings together two DNA molecules). Cohesins (often in combination with CTCF) also participate in various *cis*-tethering events including transcriptional regulation via looping and chromosome condensation through intramolecular looping such that cohesins can associate with DNA throughout the genome and in a site-specific manner (Kang et al., 2015; Poterlowicz et al., 2017; Phillips-Cremins et al., 2013; Rao et al., 2014; de Wit et al., 2015; Guo et al., 2015; Tang et al., 2015; Hansen et al., 2017; Dorsett, 2016; Kawauchi et al., 2016; Watrin et al., 2016). Formation of both *cis-* and *trans*-DNA tethers throughout the cell cycle has hampered efforts to understand the molecular etiology of cohesinopathies. For instance, work from various model systems strongly suggest that CdLS arises through transcriptional dysregulation that involve mostly *cis*-DNA tethers formed during the G1 portion of the cell cycle. In contrast, a predominant view is that RBS arises through *trans*-tethering defects that result in mitotic failure and loss of progenitor stem cells through apoptosis (Mönnich et al., 2011; Morita et al., 2012; Percival et al., 2015). More recent evidence, however, is consistent with an emerging model that transcriptional dysregulation may underlie RBS as well as CdLS such that mitotic failure is present but not a causative agent of RBS (Banerji et al., 2016; Xu et al., 2013, 2014).

The zebrafish regenerating caudal fin is a valuable model system for studies related to skeletal morphogenesis (Ton and Iovine, 2013a; Pfefferli and Jaźwińska, 2015). The fin consists of 16-18 bony fin rays, each comprising bony segments flanked by fibrous joints. The tissue itself is relatively simple, with an epidermis surrounding two hemi-rays of bone matrix that in turn surround a mesenchyme that includes blood vessels, undifferentiated fibroblasts and nerves. Upon amputation, the fin regenerates rapidly via the establishment of a proliferative compartment called the blastema.

Because gene knockdown does not require systemic treatment, evaluating gene function in the regenerating fin eliminates any potentially confounding effects of embryonic lethality upon cohesion gene knockdowns (Mönnich et al., 2011; Morita et al., 2012). Previously, we reported on a novel regenerating fin model of RBS and documented the role of *esco2* in skeletal and tissue regrowth (Banerji et al., 2016). Importantly, that study revealed that Esco2 is critical for *connexin43* (*cx43*) expression. Cx43 comprises gap junctions which confer direct communication between cells through channels that allows small signaling molecules (<1000 Da) to pass (Goodenough et al., 1996). CX43 function is conserved among vertebrates, is the most abundant connexin in bone cells, and is important for skeletal development such that *CX43* mutations lead to the skeletal disorder oculodentodigital dysplasia (ODDD) in humans and mice (Paznekas et al., 2003; Flenniken et al., 2005; Jones et al., 1993). In zebrafish, hypomorphic mutations in *cx43* cause the *short fin* (*sof ^b123^*) phenotypes, which include reduced fin length, reduced bone segment length, and reduced cell proliferation (Iovine et al., 2005). Here, we provide evidence that *smc3* knockdown recapitulates both *esco2* and *cx43* knockdown phenotypes (i.e. reduced fin and bone segment length). Critically, *smc3* is required for *cx43* expression. Moreover, we mapped Smc3 binding within the *cx43* promoter, consistent with the model that Smc3 directly impacts *cx43* expression. These studies provide proof-of-concept for a model suggesting that Esco2 activated Smc3 binds to clinically relevant skeletal regulatory genes.

## RESULTS

### Expression of *smc3* in the regenerating fin

**Fig. 1.**
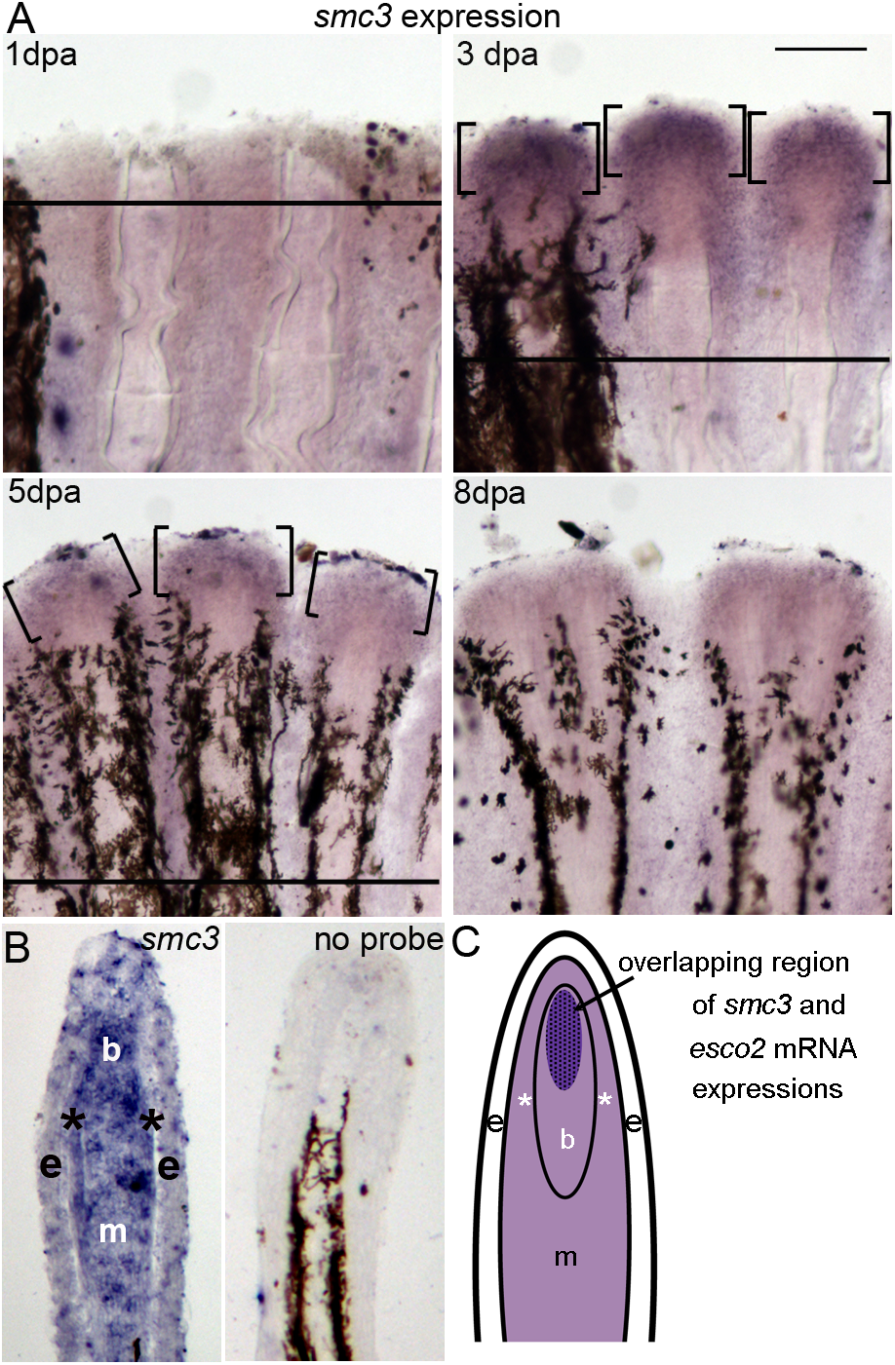
**Expression of *smc3* in whole-mount and cryosectioned regenerating fins.** (A) Expression of *smc3* by whole-mount *in situ* hybridization at various time points (1, 3, 5 and 8 dpa) (*n*=6 per timepoint). A solid line indicates the amputation plane, except in 8 dpa, where it is out of the field of view. Brackets identify regions of *smc3* expression. Scale bar: 50 µm. (B) *In situ* hybridization on a longitudinal cryosection of a 3 dpa fin showing the tissue-specific localization of *smc3* mRNA. Expression is observed in most compartments of the regenerating fin, and appears to be localized strongly in the blastemal compartment (b) with moderate expression in the epidermis (e) and proximal mesenchyme (m), including the skeletal precursor cells (*). The no probe control (right panel) shows no expression of *smc3*. Melanocytes are observed in the lateral mesenchyme. The amputation plane is out of the field of view. Three independent trials were performed with different fin sections from three different fins. (C) Schematic representation of a longitudinal section of a 3 dpa regenerating fin showing the overlapping expression patterns of *esco2* and *smc3* mRNA. Lighter purple areas indicate regions of *smc3* expression and the dark purple area represents both, *smc3* and *esco2* expression.

Esco2 is a critical regulator of fin skeletal and tissue regeneration that is required for expression of the developmental signaling factor *cx43* (Banerji et al., 2016). While Esco2 is essential for modifying the cohesin subunit Smc3 to produce sister chromatid tethering and high fidelity chromosome segregation, a role for Smc3 in mediating Esco2-dependent RBS-like skeletal and tissue defects remains unknown. To address this gap in knowledge, we evaluated *smc3* expression and function during fin regeneration. First, we completed *in situ* hybridization to monitor the temporal expression of *smc3* mRNA in 1, 3, 5 and 8 days postamputated (dpa) fins. The results reveal that *smc3* mRNA is strongly expressed at 3 dpa, similar to *esco2* expression (Fig. 1A). *smc3* expression decreased by 5 dpa and was negligible by 8 dpa (Fig. 1A). Thus, the *smc3* expression mirrors that of *esco2*, peaking in expression at 3 dpa when regeneration is at its peak (Banerji et al., 2016; Lee et al., 2005; Hoptak-Solga et al., 2008).

Expression of *esco2* mRNA is localized to the highly proliferative blastemal compartment of the fin (Banerji et al., 2016). To test whether *smc3* expression is localized similarly to the blastema, we performed *in situ* hybridization on 3 dpa cryosectioned fins. The results reveal that the expression of *smc3* correlates with *esco2* localization (Fig. 1B,C), but that *smc3* also extends to the epidermis, mesenchyme and skeletal precursor cells (Fig. 1B, left panel). No staining was detected in 3 dpa cryosectioned fins in the absence of the *smc3* probe (Fig. 1B, right panel). In combination, our studies reveal that *smc3* expression temporally and, in part, spatially coincides with that of *esco2* expression, consistent with a requirement during the early stage of regeneration specifically in the proliferative blastemal compartment of the regenerating fin.

### Knockdown of *smc3* results in reduced regenerate length, segment length and cell proliferation

**Fig. 2.**
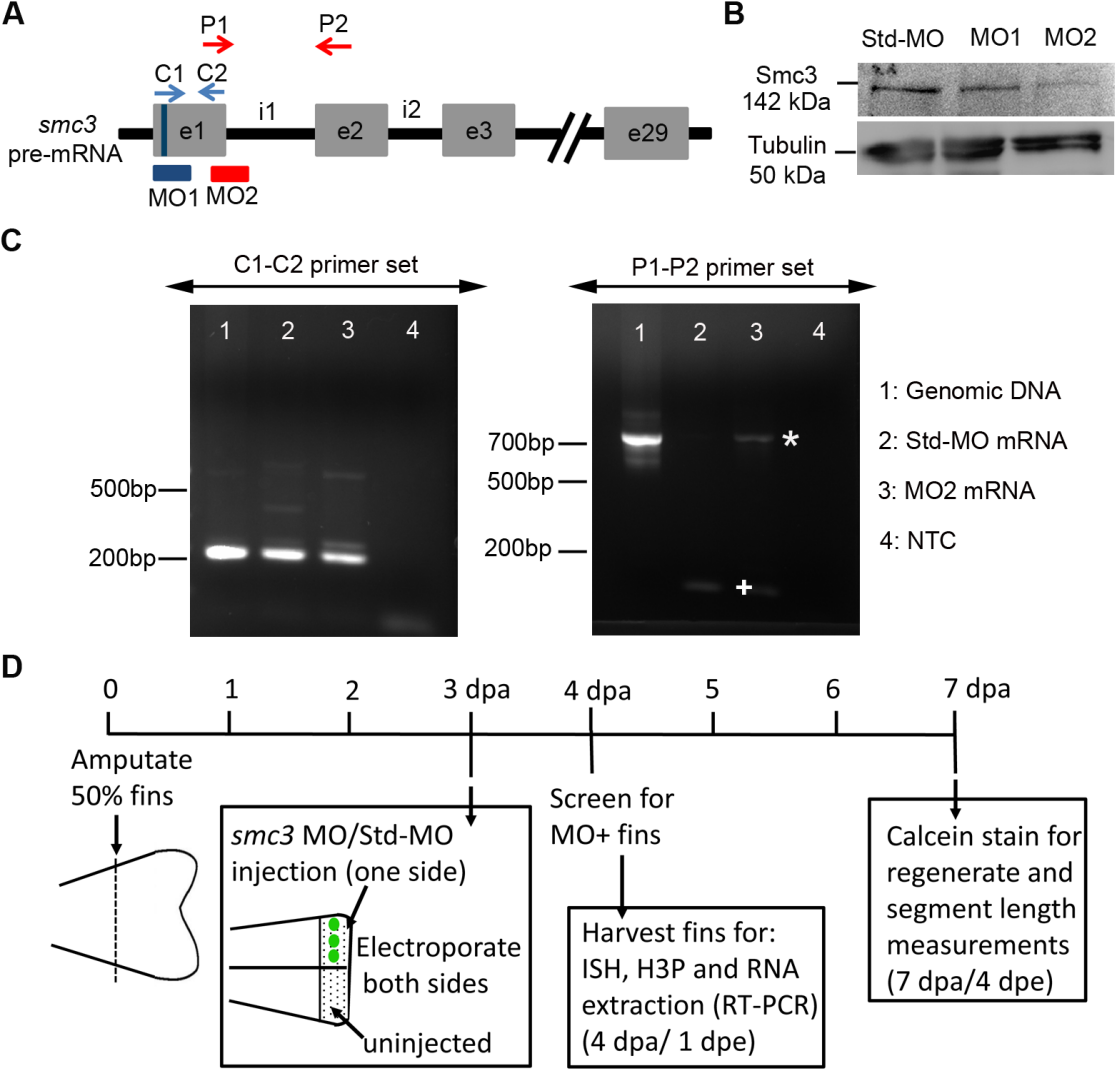
**Validating the efficiency of *smc3* MOs.** (A) Schematic representation of the zebrafish *smc3* pre-mRNA with exons (e) represented by grey boxes and the regions between the exons the introns (i). The position of MO1 (ATG blocker) at the start codon of the *smc3* gene is indicated by a blue bar (indicated on e1 with a vertical line). MO2 is positioned at the first exon and intron junction of the splice donor site (e1i1). The positions of the control primer pairs (C1-C2) are indicated with blue arrows, whereas the position of the target primer pairs (P1-P2) is indicated with red arrows. (B) Western blot analysis detects Smc3 at a predicted size of 142 kDa. Smc3 protein levels are reduced in both MO1 (62%) and MO2 (83%) fin lysates (lanes 2 and 3, respectively) compared to the Std-MO injected fin lysate (lane 1). Tubulin was used as a loading control at a predicted size of 50 kDa. Similar findings were observed in each of three trials (*n*=10 fins per trial). (C) Results of RT-PCR analysis using CI-C2 and P1-P2 primer pairs for verifying the efficiency of MO2. The templates for both these primer pairs are numbered from 1 to 4 as follows: (1) genomic DNA extracted from regenerating fins, (2) cDNA from fins injected with Std-MO, (3) cDNA from fins injected with MO2 and (4) no template control (NTC). We used three fins to generate genomic DNA and 10 fins to generate cDNA. The C1-C2 primer pair amplified an expected 210 bp product. In contrast, the P1-P2 pair amplified a 729 bp product in lane 3 (marked with *) due to the inclusion of intron1 (as predicted for the MO2-injected sample) compared to lane 2 (marked with +), which amplified the spliced product (as expected for the Std-MO injected sample). (D) Schematic outline of knockdown experiments. Fins are amputated (50% level) and permitted to regenerate for 3 days. At 3 dpa, either *smc3* MOs (MO1 and MO2) or Std-MO was microinjected to one half of the regenerating fin keeping other half uninjected. This was immediately followed by electroporation on both injected and uninjected sides of the fin. The next day, i.e. 1 dpe or 4 dpa, the injected part of the fins were evaluated for MO uptake using a fluorescence microscope. Only those fish that showed a strong signal of the fluorescein-tagged MO were used for further experiments. For experiments such as *in situ* hybridization (ISH), H3P and RNA extraction for RT-PCR, the fins were harvested at 1 dpe or 4 dpa. Note that for RNA extraction, all fin rays across the fin were injected with MO and electroporated before harvesting. For regenerate length and segment length measurement and analysis, fins were allowed to regenerate for longer period and were calcein stained at 4 dpe or 7 dpa. For each experiment *n*=8 per trial and at least three independent trials were performed.

We previously reported that Esco2 is essential for regenerate length, segment length and cell proliferation in regenerating fins (Banerji et al., 2016). Similar to *esco2*, *smc3* is essential. This precludes the use of zygotic mutants to define gene function during adult regeneration. Therefore, we designed two independent non-overlapping morpholinos (MOs) that target Smc3: one targeting the *smc3* ATG (MO1) and the second targeting the first splice site junction (exon1-intron1; e1i1) of *smc3* (MO2) (Fig. 2A). Thus, MO1 blocks the translation of Smc3 whereas MO2 alters the proper splicing of *smc3* pre-mRNA. All results were compared to a standard negative control MO (Std-MO) as previously described (Banerji et al., 2016; Bhadra and Iovine, 2015).

We first validated the efficiency of the two *smc3* MOs (MO1 and MO2) by monitoring Smc3 protein levels in fins treated with MO1, MO2 or Std-MO. The results reveal that the Smc3 protein levels were significantly reduced in the Smc3 knockdown (MO1 and MO2) lysates compared to the Std-MO control lysates (Fig. 2B). To confirm the effectiveness of MO2 to block proper splicing, we performed reverse transcription polymerase chain reaction (RT-PCR). RT-PCR results revealed that intron1 was retrieved only when fins were injected with MO2 and not when injected with Std-MO (Fig. 2C). Sequencing confirmed that the products represent the *smc3* gene (not shown). These analyses provide strong evidence for target specificity for both MO1 and MO2 (Eisen and Smith, 2008).

**Fig. 3.**
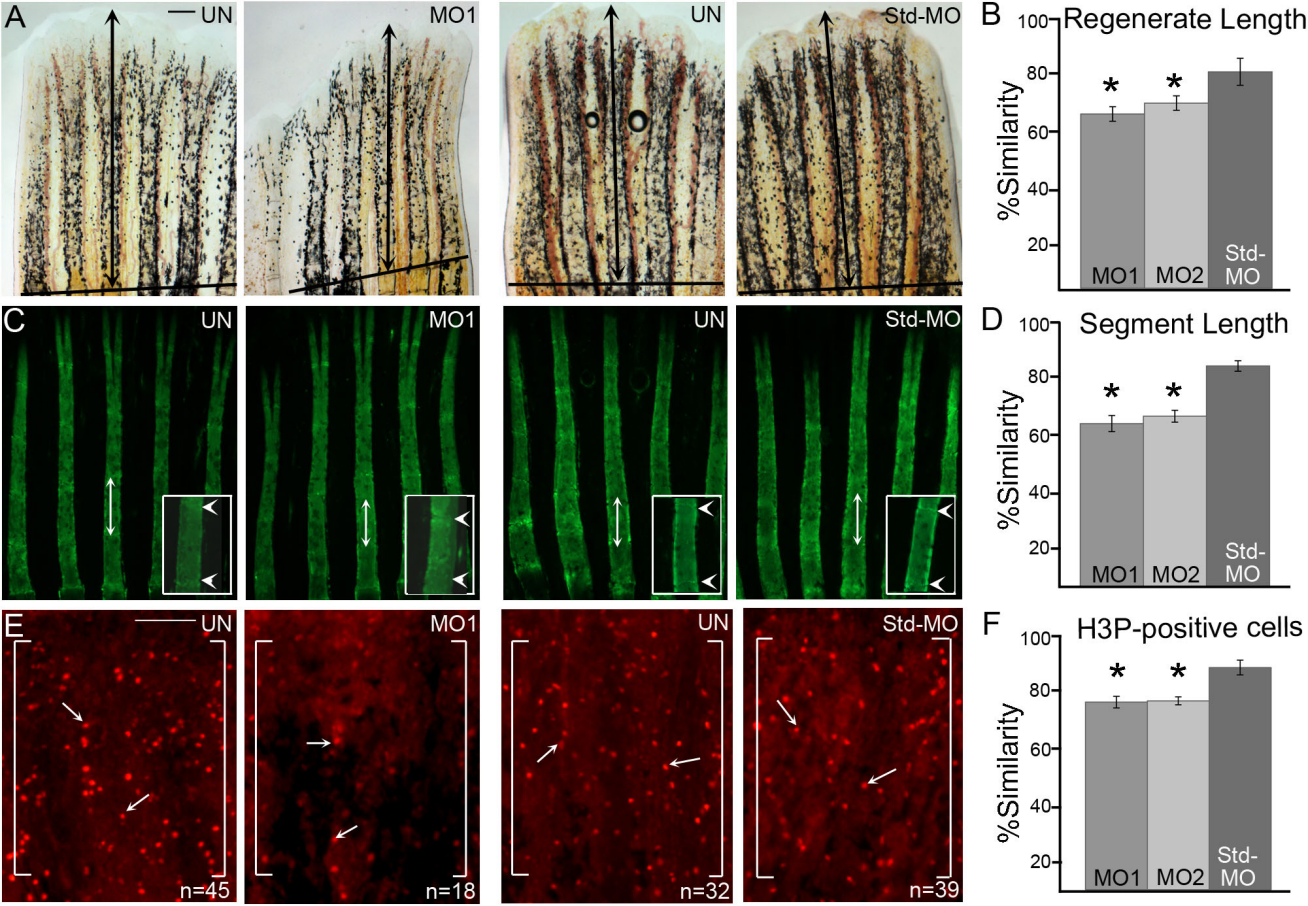
**MO-mediated *smc3* knockdown results in reduced regenerate length, segment length and cell proliferation.** (A) Representative images of uninjected (UN), *smc3* MO-injected (MO1) and Std-MO injected fins. Total regenerate length was calculated by measuring the distance between the amputation plane (indicated by a solid black line) to the distal end of the third fin ray (black arrows indicate the length measured). (B) Graph shows the significant reduction (indicated by *) of regenerate length in *smc3* knockdown fins (for both MO1 and MO2) compared to the Std-MO injected fins using the percent similarity method. (C) Representative images of calcein stained fins of uninjected (UN), *smc3* MO injected (MO1) and Std-MO injected fins. Segment length was calculated by measuring the distance between first two joints in the 3rd fin ray (black arrows indicates the length measured). Higher magnification images of segments are shown with joints indicated by white arrowheads. (D) Graph shows that significant reduction (indicated by *) of segment length in *smc3* knockdown (for both MO1 and MO2) compared to Std-MO injected fins using the percent similarity method. (E) Representative images of H3P-positive cells in uninjected (UN), *smc3* MO injected (MO1) and Std-MO injected fins. Measurements were taken from the distal most 250 μm of the 3rd ray. White brackets mark the defined area and *n* represents the number of H3P-positive cells in that area. Arrows identify H3P-positive cells. (F) Graph shows the significant reduction (indicated by *) in the number of H3P-positive cells in *smc3* knockdown (for both MO1 and MO2) compared to Std-MO injected fins using the percent similarity method. For each experiment *n*=8 fins per trial and three independent trials were performed. *P<0.05, two tailed unpaired Student's *t*-test. Data are mean±s.e.m. Scale bars: 50 µm in A; 100 µm in E.

Using both MOs we carried out microinjection and electroporation as previously described (Govindan et al., 2016; Banerji et al., 2016) (Fig. 2D). All MOs are tagged with fluorescein, allowing us to validate cellular uptake microscopically 1 day postelectroporation (dpe) or 4 dpa (Ton and Iovine, 2013b). All MO-positive fins were selected for further experiments, while MO-negative fins were excluded (i.e. these fins likely represent failed electroporation). For measurement of regenerate length and segment length, *smc3* knockdown/Std-MO fins were calcein stained at 4 dpe/7 dpa and measured. To reduce the effect of fin-to-fin variation, we utilized the percent similarity method in which values close to 100% indicates no difference between injected and non-injected sides of the same fin. Values less than 100% indicate reduced growth of the injected fin side compared to the non-injected side of the same fin, whereas values greater than 100% indicate increased growth of the injected fin side compared to the non-injected side (Govindan et al., 2016; Bhadra and Iovine, 2015; Banerji et al., 2016). Quantification of regenerate length was based on the distance from the plane of amputation to the distal end of the 3rd fin ray. Quantification of bone segment length was based on measurements obtained from the first segment distal to the amputation plane of the 3rd fin ray. The Std-MO injected fins showed a high percentage similarity to the uninjected side, indicating that the control MO had no effect on regenerate and bone segment length as expected. In contrast, both MO1 and MO2 showed low percentage of similarities, indicating significantly reduced growth for both regenerate length and segment length in injected fins compared to internal controls of the non-injected sides of the same fins (Fig. 3A-D; Fig. S1).

Esco2 knockdown also results in reduced cell proliferation but not elevated levels of apoptosis (Banerji et al., 2016). Thus, we next addressed whether the effect of *smc3* knockdown on both regenerate length and segment length was based on altered levels of either cell proliferation or apoptosis. To test the first of these possibilities, we quantified the number of mitotic cells by staining for Histone-3 phosphate (H3P) on 1 dpe *smc3* knockdown (MO1 and MO2) and Std-MO injected fins. The results reveal significant reduction in H3P-positive cells in *smc3* knockdown fins compared to the control fins (Fig. 3E,F; Fig. S1). We then tested the possibility that apoptosis or programmed cell death (PCD) is increased in Smc3 depleted fins. TUNEL assays were performed on fins injected with either *smc3* MO1 or Std-MO in one half of the fin, keeping the other half uninjected. Fins were harvested at 1 dpe/4 dpa for TUNEL staining. The results failed to reveal any statistically significant difference in the number of apoptotic cells between the MO1 injected and Std-MO injected fins (Fig. S2). Thus, Smc3-dependent regeneration defects in reducing cell proliferation but not elevating PCD are similar to those previously reported for Esco2 (Banerji et al., 2016). Having validated *smc3*-knockdown phenotypes (reduced regenerate length, segment length and cell proliferation) using two non-overlapping MOs, all subsequent experiments were performed using a single targeting *smc3*-MO (MO1).

### *smc3* and *esco2* function together during skeletal regeneration

**Fig. 4.**
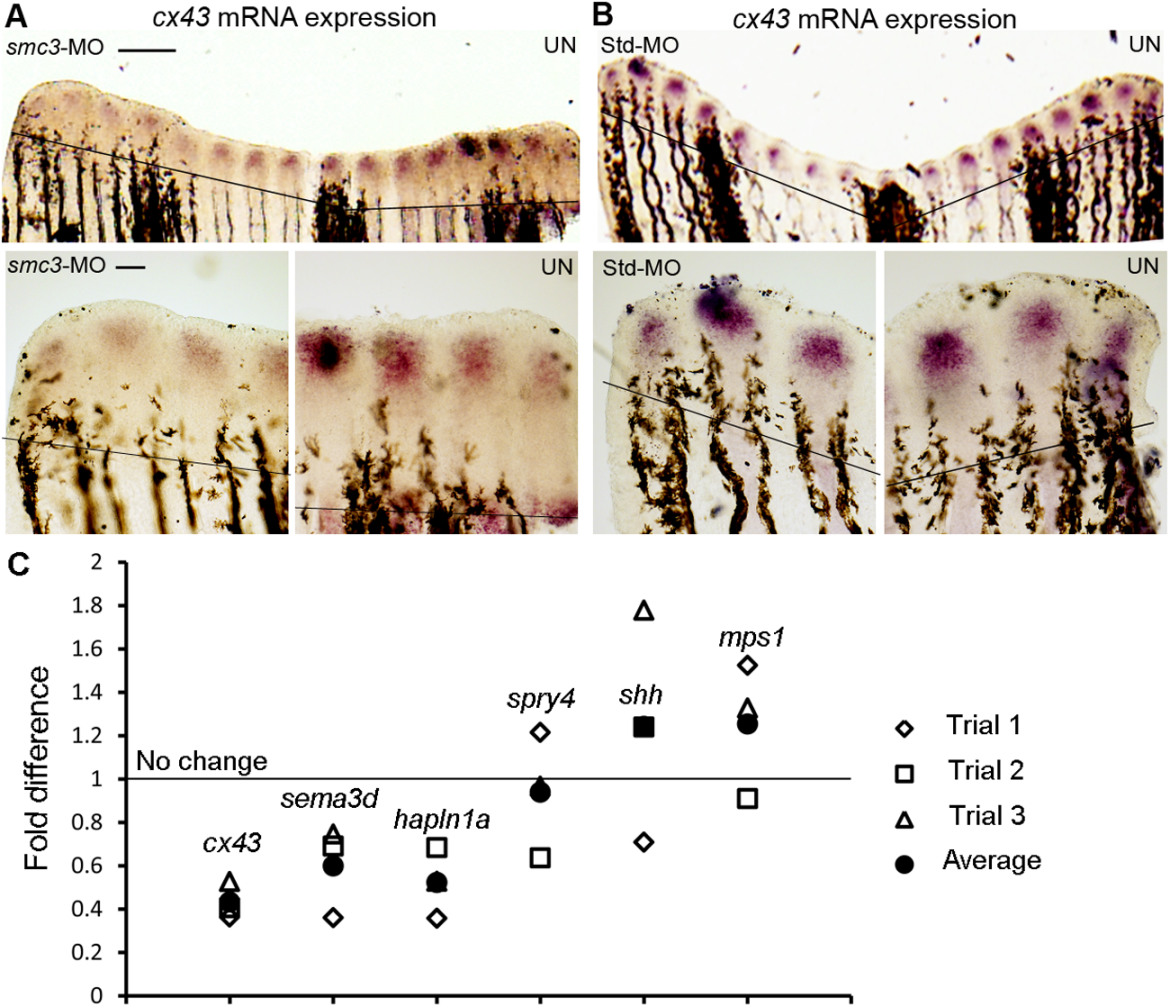
***smc3* regulates the expression of *cx43* in regenerating fins.** (A) Representative image of a fin with the *smc3*-knockdown side (*smc3*-MO) showing decreased *cx43* staining compared to the uninjected side (UN). Higher magnification of the *smc3* knockdown side of the same fin shows reduced levels of *cx43* expression compared to the uninjected side), which shows normal *cx43* levels. (B) Representative image of Std-MO-injected fin revealing similar *cx43* levels in both injected and uninjected sides. Higher magnification of the same fin shows normal and similar levels of *cx43* expression in both injected and uninjected sides (UN). For this experiment *n*=6 fins per trial and three independent trials were performed. The solid line indicates the amputation plane. Scale bars: 100 µm in the upper panel; 50 µm in the lower panel. (C) qPCR confirms the downregulation of *cx43* and *cx43*-dependent target genes (*sema3d* and *hapln1a*) following Smc3 knockdown. Fold difference values from the qPCR are shown; a fold difference of 1 is considered as no change with respect to Std-MO-injected fins (indicated by the horizontal line). Three independent Smc3 knockdown samples were prepared. Each sample was tested in duplicates (trials 1-3) for *cx43*, *hapln1a*, *sema3d*, *shh*, *spry4* and *mps1* (compared to the internal reference gene, *actin*). Each of the three trials are denoted by open shapes and the averages are denoted by solid circles.

*esco2* is critical for *cx43* expression, although the basis for this regulation remains unknown (Banerji et al., 2016). Thus, it became important to determine if *smc3*-knockdown also influences *cx43* expression. We performed whole-mount *in situ* hybridization with *cx43* probe on *smc3* knockdown fins. Half of the fin was injected with MO1 or Std-MO and the other half was uninjected as an internal control. The *smc3* knockdown side exhibited significantly reduced expression of *cx43* compared to the uninjected side (Fig. 4A). In contrast, the Std-MO injected side showed no difference in *cx43* expression compared to the uninjected side (Fig. 4B). Because reduced cell proliferation is not sufficient to reduce *cx43* expression (Govindan and Iovine, 2014; Bhadra and Iovine, 2015), the observed reduction of *cx43* expression in *smc3* knockdown fins is likely not the result of reduced cell proliferation.

To complement these studies, we next completed quantitative RT-PCR (qPCR) to confirm that *cx43* expression is reduced following *smc3* knockdown (Fig. 4C and Table 1; primers in Table S1). Importantly, we found that *cx43*, in addition to its downstream target genes *sema3d* and *hapln1a* (Ton and Iovine, 2012; Govindan and Iovine, 2014), are reduced following *smc3* knockdown. Moreover, we found that expression of *mono polar spindle* (*mps1*), *sonic hedgehog (shh*) and *sprouty4* (*spry4*) (Poss et al., 2002; Laforest et al., 1998; Lee et al., 2005) are not reduced in *smc3* knockdown fins. Together, these findings are remarkably similar to our prior findings regarding changes in *cx43* and downstream gene expressions in fins knocked down for *esco2* (Banerji et al., 2016).

**Table 1.**
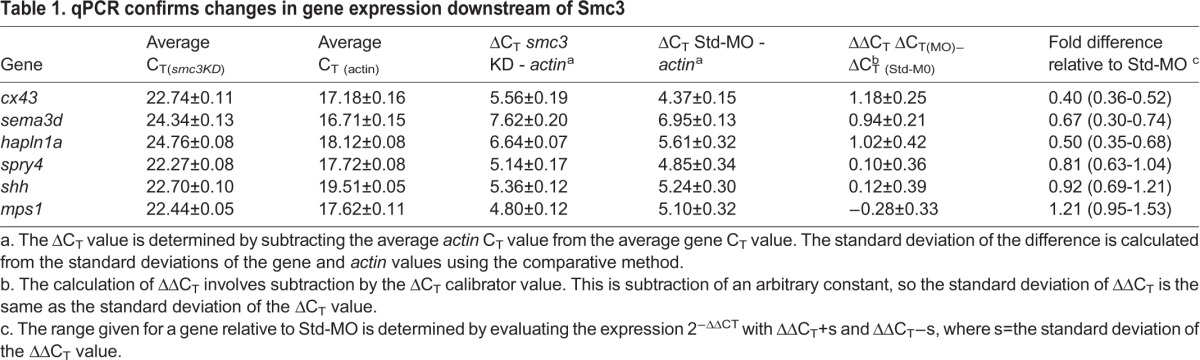
**qPCR confirms changes in gene expression downstream of Smc3**

**Fig. 5.**
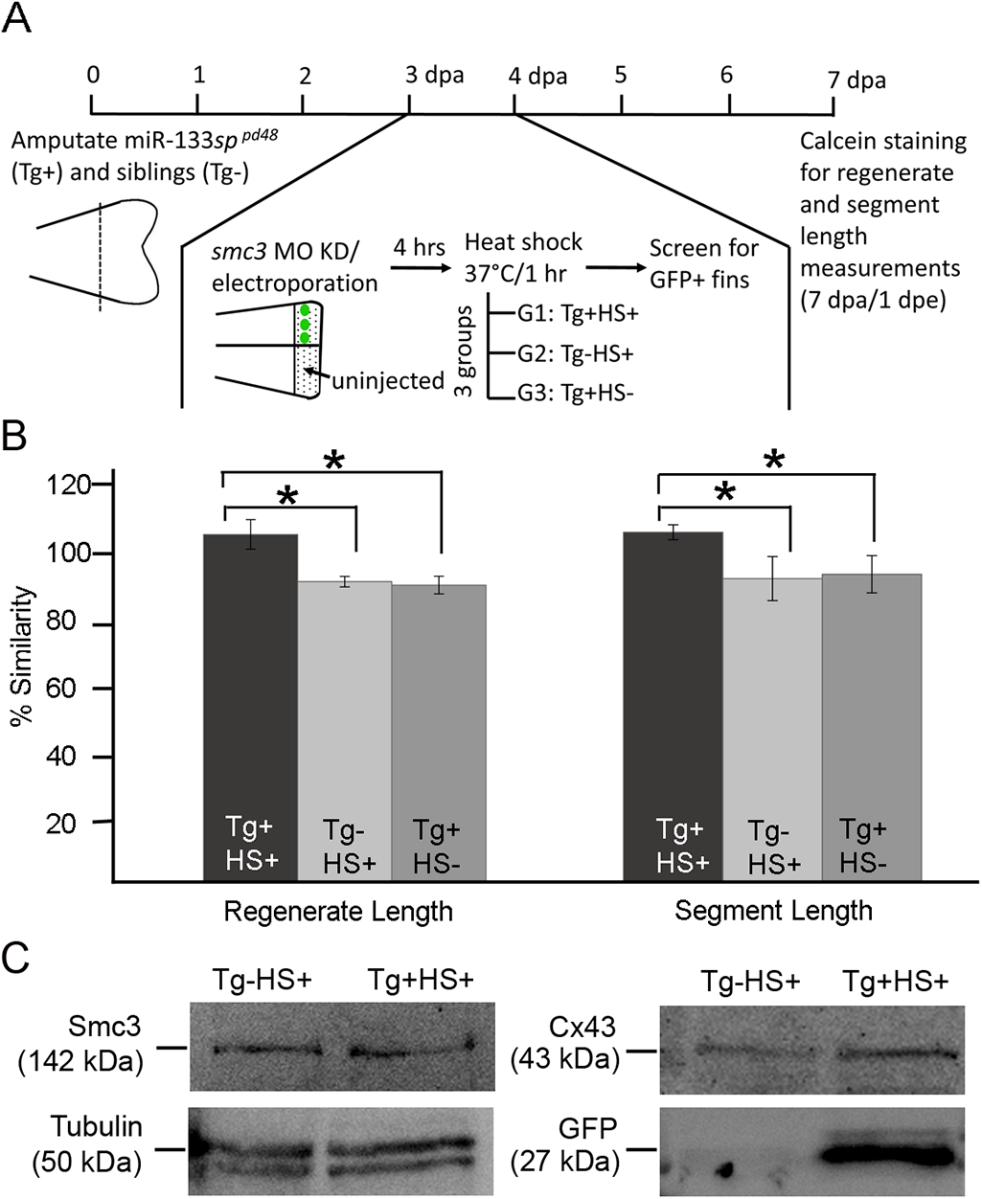
**Overexpression of *cx43* rescues *smc3*-dependent skeletal phenotypes.** (A) Experimental timeline providing details of the fin amputation, MO injection/electroporation, heat shock and data analysis process. Fin amputation (50% level) was performed on transgenic *hsp70:miR-133sp^pd48^* fish (Tg+) and their siblings (Tg−). At 3 dpa, *smc3* MO was injected in one half of the fin keeping the other half uninjected. This step was immediately followed by electroporating both sides of the fin. After a period of 4 h, the heat shock process began. At this point there were three groups of fish: (1) Tg+HS+, the transgenic-positive fish that were heat shocked at 37°C for 1 h; (2) Tg+HS−, the transgenic-positive fish but were not heat shocked; (3) Tg−HS+, the siblings (transgenic-negative) that were similarly heat shocked as Tg+HS+. At 4 dpa or 1 dpe, the Tg+HS+ fins were screened for positive GFP expression, which indicated transgene induction. The control groups (Tg+HS− and Tg−HS+) were GFP negative, indicating absence of transgene induction. For regenerate length and segment length measurement and data analysis, fins were calcein stained at 7 dpa or 4 dpe. (B) The graph reveals significant rescue of *smc3*-dependent regenerate and segment length defects in Tg+HS− *smc3* knockdown fins compared to the control groups (Tg−HS+ and Tg+HS−). For each experiment *n*=8 fins per trial and three independent trials were performed. *P<0.05, two tailed unpaired Student's *t*-test. Data are mean±s.e.m. (C) Smc3 protein expression is nearly similar (90%) in the Tg-HS+ (lane 1) and Tg+HS+ (lane 2) fin lysates (normalized to Tubulin). In contrast, Cx43 protein is increased) in Tg+HS+ (lane 2) fin lysate compared to Tg−HS+ (lane 1) fin lysate, as expected. Similarly, GFP protein expression is also increased in Tg+HS+ fin lysate (lane 2) compared to Tg−HS+ fin lysate. Tubulin (50 kDa) was used as the loading control for all blots. ImageJ software was used for analysis of relative band intensity.

To provide further evidence that *smc3* acts upstream of *cx43***,** we tested for rescue of *smc3-*MO phenotypes by overexpressing Cx43 (Banerji et al., 2016). For this purpose, we used the transgenic line *Tg*(*hsp70:miR-133sp^pd48^*), which overexpresses Cx43 in both regenerating heart and fins. In this line, heat shock induces expression of the miR-133 target sequence fused to EGFP and therefore sequesters the *miR-133*. This causes increased expression of miR-133 target genes such as *cx43* (Yin et al., 2012; Banerji et al., 2016). We tested three groups of fish, as follows: (1) transgene positive and heat shocked (Tg+HS+), (2) transgene negative and heat shocked (Tg−HS+) and (3) transgene positive but not heat shocked (Tg+HS−) (Fig. 5A). Importantly, three independent heat shock trials revealed that both regenerate length and bone segment length defects otherwise exhibited in *smc3* knockdown were significantly rescued in the Tg+HS+ group (Fig. 5B). This rescue was specific to transgene activation and was not induced by heat shock alone or in combination with any other group. We previously confirmed up-regulation of both *cx43* mRNA and Cx43 protein levels in Tg+HS+ fins and also demonstrated that the *esco2* mRNA and Esco2 protein levels are comparable between the Tg+HS+ and Tg−HS+ fins (Banerji et al., 2016). Similarly, to rule out the possibility that the transgene induces Smc3 expression, we further confirmed that Smc3 protein is not upregulated in Tg+HS+ fins compared to the Tg−HS+ fins. (Fig. 5C). These findings support an exciting model that Esco2 and Smc3 function together upstream to regulate *cx43* gene expression.

**Fig. 6.**
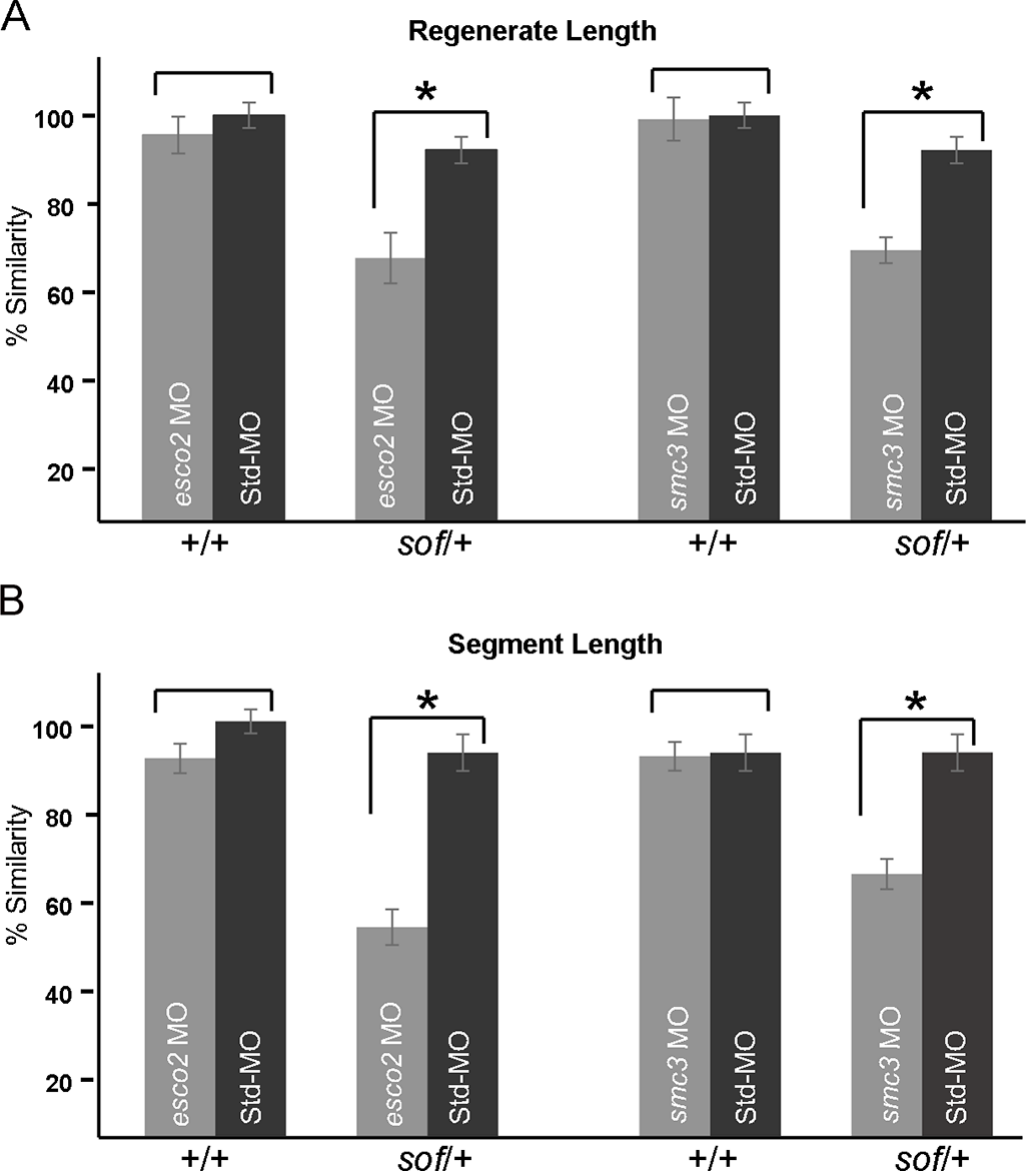
**Synergy experiments demonstrate that both *esco2* and *smc3* act in a common pathway with *cx43*.** (A) The graphs representing percent similarities show that the subthreshold doses of *esco2* MO (0.5 mM) and *smc3* MO (0.5 mM) do not cause significant reduction in regenerate length of wild-type fins (+/+) when compared to Std-MO (0.5 mM) injected into wild-type fins (+/+). The graphs representing percent similarities show that the subthreshold dose of both MOs (*esco2* and *smc3*) significantly reduces regenerate length when injected in *sof* heterozygotes (*sof*/*+*), compared with Std-MO (0.5 mM). (B) The graphs representing percent similarities show that the subthreshold doses of *esco2* MO (0.5 mM) and *smc3* MO (0.5 mM) do not cause significant reduction in segment length of wild-type fins (+/+) when compared to Std-MO (0.5 mM) injected into wild-type fins (+/+). The graphs representing percent similarities show that the sub-threshold dose of both MOs (*esco2* and *smc3*) significantly reduce segment length when injected in *sof* heterozygotes (*sof*/*+*), compared with Std-MO (0.5 mM). For each experiment *n*=8 fins per trial and 3 independent trials were performed. *P<0.05, two tailed unpaired Student's *t*-test. Data are mean±s.e.m.

Although rescue using *Tg*(*hsp70:miR-133sp^pd48^*) supports our model that *cx43* is functionally activated downstream of Esco2 and Smc3, because *miR-133* has multiple targets (Yin et al., 2008), we cannot rule out the possibility that a different target gene is responsible for the rescue. Therefore, to complement these studies we tested for synergistic interactions between *esco2* and *cx43*, and between *smc3* and *cx43*. First, we identified doses of the *esco2* and *smc3* MOs that alone did not cause skeletal phenotypes when compared to the standard control MO. We found that MO concentrations of 0.5 mM for both *esco2* and *smc3* were insufficient to cause skeletal defects (Fig. 6). Next, we injected these subthreshold doses of either the *esco2* MO or the *smc3* MO into regenerating fins of *sof* heterozygotes (*sof*/*+*), which carry a hypomorphic mutation in *cx43* (Iovine et al., 2005). The growth and regeneration of *sof*/*+* fins are only marginally shorter than wild-type fins and therefore represent a subthreshold activity of *cx43* function. Remarkably, injection of subthreshold levels of *esco2* MO significantly reduced regenerate and bone segment growth in *sof*/+ fins, compared to wild-type fins (Fig. 6). Moreover, injection of subthreshold levels of *smc3* similarly reduced regenerate and bone segment growth in *sof*/+ fins, compared to wild-type fins (Fig. 6). These findings provide compelling evidence of synergy and demonstrate that *esco2* and *smc3* act in a common genetic pathway with *cx43*.

### Smc3 directly binds to a specific region of the *cx43* promoter

What is the basis through which both Esco2 and Smc3 regulate *cx43* expression? To address this issue, we switched to a less complex AB9 fibroblast cell line previously reported to complement *in vivo* regenerating fin studies and express Cx43 (Bhadra et al., 2015). AB9 cells are primary fibroblasts derived from regenerating caudal fins of the adult zebrafish. We first tested whether AB9 cells also express Esco2 and Smc3. AB9 cells grown on a coverslip were fixed and processed for immunofluorescence. The results show that anti-Esco2 antibody and anti-Smc3 antibody both overlap with the DAPI-stained nuclei, revealing that both Esco2 and Smc3 are located in cell nuclei (Fig. S3). Having validated the AB9 cell system, we next tested whether either *esco2* or *smc3* similarly regulate Cx43 protein levels as occurs in regenerating fins. Cx43 protein levels were monitored by western blotting in AB9 cells knocked down for either *esco2* MO or *smc3* MO. The results show that Esco2 or Smc3 proteins were each reduced using their respective knockdown morpholinos (Fig. S3). Esco2 is reduced by ∼65%, and Smc3 is reduced by ∼60%. Critically, Cx43 protein levels also were reduced following knockdown with either MO (Fig. S2). Cx43 is reduced by 92% following Esco2 knockdown, and is reduced by about 68% following Smc3 knockdown. Therefore, this tissue culture AB9 system recapitulates the reduced Cx43 protein levels upon Esco2 and Smc3 knockdowns in regenerating fins (Banerji et al., 2016).

**Fig. 7.**
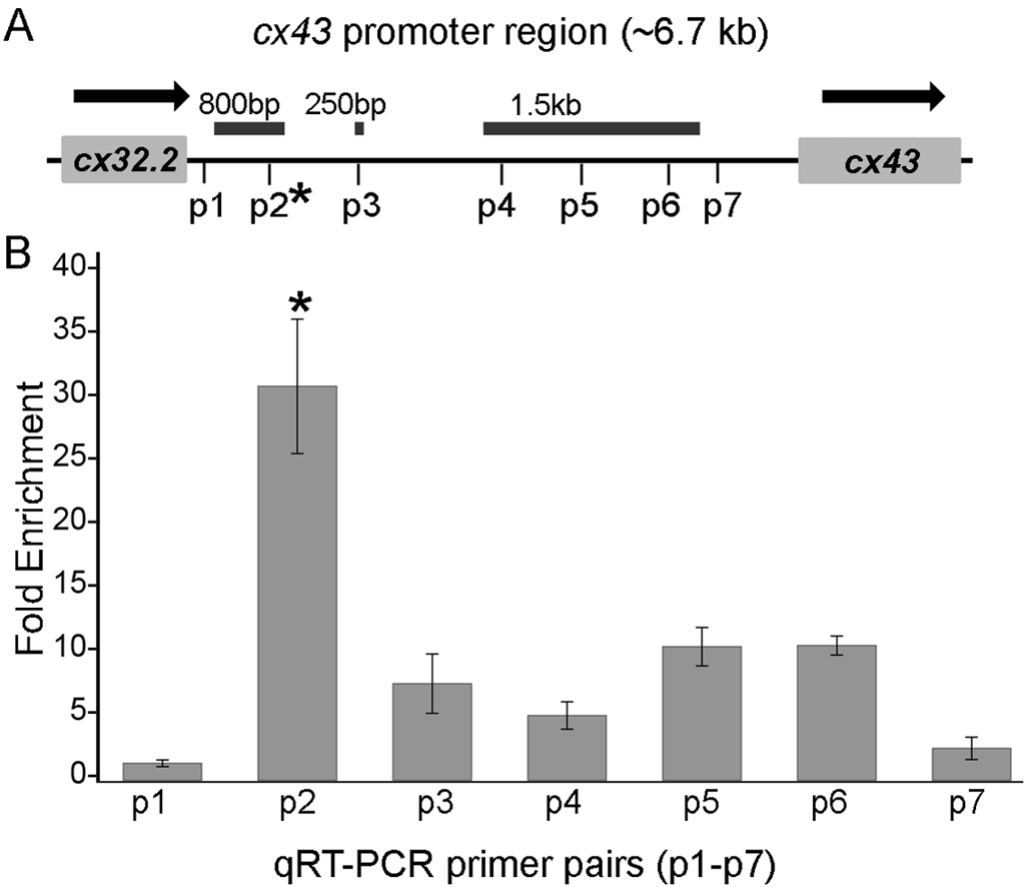
**Smc3 binds at a specific location of the *cx43* promoter.** (A) Schematic representation of the zebrafish *cx43* promoter. It is ∼6.7 kb in length, adjacent to an additional connexin gene (*cx32.2*). The horizontal bars indicate the binding regions of Smc3 inferred from qualitative PCR results. The positions of the seven qRT-PCR primer pairs (p1-p6) are indicated on the promoter region. The two primer pairs (p1 and p7) are the negative controls, since they lie at a region not predicted from previous PCR results. (B) The graph represents the fold enrichment of Smc3 binding (normalized to IgG) at different regions of the *cx43* promoter. Significant enrichment was observed at p2 location of the promoter suggesting positive binding of Smc3 at the p2 region. *P<0.001, one-way ANOVA with Tukey's multiple comparison post hoc test. Data are mean±s.e.m. from three independent trials.

It is well established that cohesins bind directly and stabilize DNA-tethering structures required for efficient gene expression (Dorsett, 2016; Merkenschlager and Nora, 2016; Jeppsson et al., 2014). Thus, we hypothesized that Smc3, as a part of the cohesin complex, directly binds to a segment of the *cx43* promoter. The *cx43* promoter is ∼6.7 kb in length, adjacent to an additional connexin gene (*cx32.2*) that resides upstream of the *cx43* coding sequence (Chatterjee et al., 2005; Fig. 7A). We assayed Smc3 binding to the *cx43* promoter by performing chromatin immunoprecipitation (ChIP) on AB9 cells. We first optimized the ChIP procedure by qualitative PCR analysis and using Smc3 as the target antibody and IgG as the negative control. We designed 31 primers pairs that, in overlapping fashion, span the entire 6.7 kb promoter (Table S2). Positive Smc3 binding was observed for primers 2-6 (800 bp), primer 11 (250 bp) and primers 18-28 (1.5 kb) (Fig. 7A). In contrast, the negative control (IgG) exhibited little to no binding throughout the promoter length.

To investigate in detail the specific regions of the *cx43* promoter to which Smc3 binds, we next performed qPCR. We designed five primer pairs that spanned the Smc3 positive binding regions obtained from our qualitative PCR analysis (p2- p6) and two primer pairs as negative controls that fall within the no binding zone (p1 and p7) (Table S3). The results reveal significant binding of Smc3 specifically within one region (p2) of the *cx43* promoter (Fig. 7B). Binding was also observed at p3-p6, but at levels that did not rise to statistically significant levels. The negative controls (p1 and p7) showed negligible binding. These ChIP results provide strong evidence that Smc3 binds directly to the *cx43* promoter.

## DISCUSSION

Esco2 mutations are the only known etiologic agent for RBS. Previously, we established *esco2* knockdown in regenerating fin as a powerful system from which to elucidate the molecular basis of RBS. One major revelation of the current study is that Smc3 functions in a similar manner as Esco2 during fin regeneration. First, *smc3* mRNA expression coincides with *esco2* expression in the proliferative blastemal compartment of the regenerating fin. Second, morpholino-mediated *smc3* knockdown revealed that Smc3-dependent phenotypes (i.e. reduced regenerate length, bone segment length and cell proliferation in the absence of increased PCD) recapitulate the *esco2*/*cx43*-dependent phenotypes. Third, we see a reduction in the *cx43* expression levels, and in *cx43* target genes, in *smc3* knockdown fins. Fourth, transgene dependent overexpression of target genes that include *cx43* rescues Smc3-dependent phenotypes to a similar degree as Esco2-dependent phenotypes. Finally, we find evidence of synergistic interactions between *esco2*, *smc3*, and *cx43*. Thus, the combination of our current and previous findings (Banerji et al., 2016) provide compelling evidence that Esco2, Smc3, and Cx43 function in a common pathway, and suggest that RBS may be a transcriptional malady similar to that of CdLS.

**Fig. 8.**
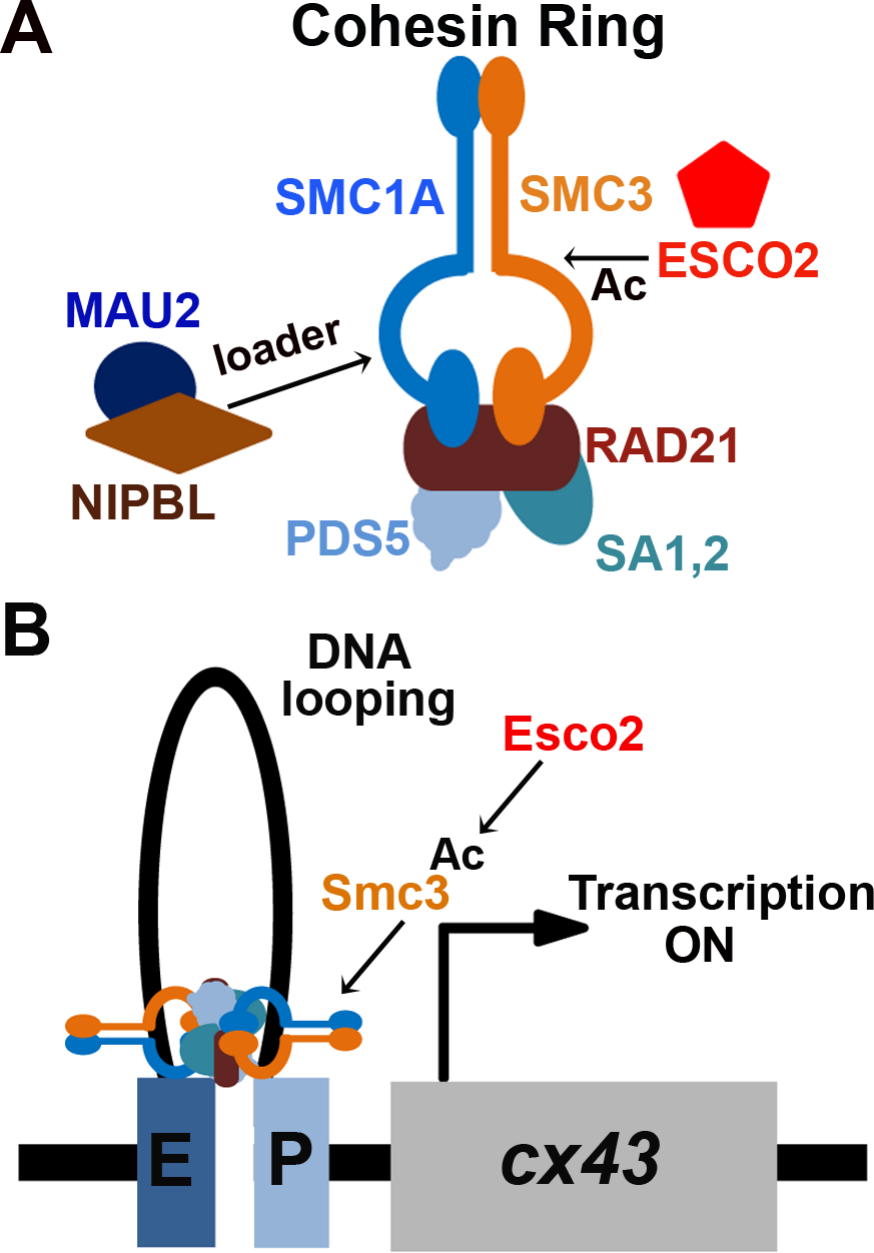
**Esco2-dependent *cis*-DNA looping model underlying the etiology of RBS.** (A) Schematic representation of the cohesin ring complex. It is composed of two structural maintenance of chromosome (SMC) subunits (SMC1A and SMC3) and three non-SMC subunits (RAD21, SA1, 2 and PDS5). The cohesin auxiliary factor, NIPBL-MAU2 heterodimer complex helps in cohesin ring opening/closing reactions that loads cohesins onto DNA. Another auxiliary factor, *ESCO2*, is a member of the *ESCO* family of N-acetyltransferases that acetylates the SMC3 cohesin subunit. (B) A model depicting the Esco2-dependent *cis*-DNA tethering mechanism underlying RBS in which the acetyltransferace Esco2 activates its target, Smc3 (denoted by Ac), which binds the *cx43* promoter, thus activating *cx43* transcription. This process is believed to occur through a *cis*-DNA looping mechanism that connects the enhancer (E) and promoter (P) of the *cx43* gene.

A popular model is that Esco2 deficiency results in mitotic failure and progenitor cell death through apoptosis. A second revelation of the current study is that RBS developmental phenotypes may instead arise directly from reduced or altered cohesin (Smc3) binding to the promoter of clinically relevant skeletal development genes. As proof-of-concept, our ChIP experiments demonstrate that Smc3 physically binds to the *cx43* promoter and is required, along with Esco2, for efficient *cx43* expression. Cx43 represents a valuable and informative target given that mutations in human *CX43* results in ODDD, and that CdLS models similarly report aberrant expression of *CX43* (Mönnich et al., 2011; Kawauchi et al., 2009). Current mechanistic models of cohesin-based regulation of gene expression indicate that cohesin stabilizes looped DNA through which distant enhancer and a proximal promoter may be brought into registration (Kang et al., 2015; Poterlowicz et al., 2017; Phillips-Cremins et al., 2013; Rao et al., 2014; de Wit et al., 2015; Guo et al., 2015; Tang et al., 2015; Hansen et al., 2017). Our results, showing that cohesin (i.e. Smc3) binds to the *cx43* promoter, combined with the established role for Esco2 in Smc3-acetylation, are consistent with a similar model in which Esco2 and Smc3 may induce expression of skeletal genes (i.e. *cx43*) through changes in chromatin architecture (Fig. 8). While speculative, this model is consistent with evidence that Esco2 functions during interphase, acetylates Smc3 and that cohesins stabilize DNA loops (Kim et al., 2008; Rahman et al., 2015; Xu et al., 2013; Mönnich et al., 2011; Leem et al., 2011; Song et al., 2012; Kang et al., 2015; Poterlowicz et al., 2017; Phillips-Cremins et al., 2013; Rao et al., 2014; de Wit et al., 2015; Guo et al., 2015; Tang et al., 2015; Hansen et al., 2017). Future studies are required to provide further support for such a model, including identification of the distant enhancer element and demonstration of DNA looping through cohesion.

Both CdLS and RBS are grouped under a similar disease category of cohesinopathies, yet the etiologies of these sister maladies are considered different. Transcriptional dysregulation is considered to be the primary mechanism underlying CdLS (Krantz et al., 2004; Tonkin et al., 2004; Gillis et al., 2004; Musio et al., 2006; Deardorff et al., 2007, 2012a,b; Zhang et al., 2009). For example, defects in *cis*-DNA tethering events result in severe to mild phenotypes observed in CdLS. Cohesin subunits (SMC1A and SMC3) and NIPBL interact with Mediator complexes along with RNA polymerase II that bring long-distance enhancers to close proximity of the promoter of transcriptionally active genes via a *cis*-mediated DNA looping event (Kagey et al., 2010). The molecular mechanism underlying RBS is thought to occur through *trans*-tethering mitotic defects. It is true that mitotic failure and modest levels of apoptotic are often accompanied in mouse and zebrafish embryo studies of RBS and our current findings do not rule out the possibility that these can contribute to developmental defects (Mönnich et al., 2011; Horsfield et al., 2012; Mehta et al., 2013; Whelan et al., 2012). However, our findings that RBS-type phenotypes (skeletal defects) can occur in the absence of apoptosis greatly diminishes these models. Instead, our data suggests a unified mechanism for both RBS and CdLS through transcriptional dysregulation (Banerji et al., 2017).

Despite the similar and overlapping phenotyopes of RBS and CdL, only cells from RBS typically exhibit mitotic failure and elevated levels of apoptosis. Although the relative absence of mitotic failure in CdLS cells led researchers to propose a transcriptional dysregulation mechanism, this conclusion failed to translate to models of RBS. Based on our findings, we suggest that changes in gene dosage is a critical aspect of both CdLS and RBS phenotypes. For instance, an elegant study performed in yeast revealed differential dosage effects on a subset of cohesion-related functions (Heidinger-Pauli et al., 2010). In humans, CdLS arises due to heterozygous dominant mutations in cohesion pathway genes. Thus, one functional copy of the gene may be sufficient to support cohesion but may not be sufficient to prevent changes in gene transcription. In contrast, RBS arises due to homozygous recessive mutations. Therefore, both copies of the *ESCO2* gene are defective, which blocks all cohesion pathway function such that mitotic defects appear more prevalent and thus obscures contributions provided by transcription dysregulation. Our studies demonstrating that Esco2 and Smc3 function together to regulate *cx43* expression provide compelling evidence for a more unified model linking the underlying mechanisms of CdLS and RBS cohesinopathies.

## MATERIALS AND METHODS

### Statement on the ethical treatment of animals

This study was performed strictly according to the recommendations in the Guide for the Care and Use of Laboratory Animals of the National Institutes of Health. Lehigh's Institutional Animal Care and Use Committee (IACUC) approved the protocols performed in the manuscript (Protocol identification # 190, approved 05/19/16). Lehigh University's Animal Welfare Assurance Number is A-3877-01. All experiments were performed to minimize pain and discomfort.

### Housing and husbandry

Zebrafish (*Danio rerio*) were housed in a re-circulating system built by Aquatic Habitats (now known as Pentair Aquatic Habitats, Apopka, FL, USA). The fish room has a 14:10 light:dark cycle with tightly regulated room temperature ranging from 27°C to 29°C (Westerfield, 1993). Monitoring of the water quality is performed automatically to maintain conductivity of 400–600 µs and pH in the range of 6.95–7.30. A biofilter is used to maintain nitrogen levels and a 10% water change occurs daily. Sequential filtration of recirculating water was carried out using pad filters, bag filters and a carbon canister before circulating over ultraviolet lights for sterilization. Fish feeding schedule was as follows: fed three times daily, once with brine shrimp (hatched from INVE artemia cysts) and twice with flake food (Aquatox AX5, Aquaneering, San Diego, CA, USA) supplemented with 7.5% micropellets (Hikari, Hayward, CA, USA), 7.5% Golden Pearl (300–500 µm, Brine Shrimp Direct, Ogden, UT, USA) and 5% Cyclo-Peeze (Argent Labs, Redmond WA, USA).

### Zebrafish strains and fin amputations

Wild-type (C32), *short fin* (*sof^b123^*) and *Tg* (*hsp70: miR-133sp^pd48^*) (Iovine and Johnson, 2000; Yin et al., 2012) *Danio rerio* animals were used. Males and females from 6 months to 1 year of age were included. All procedures involving caudal fin amputations, fin regeneration, and harvesting were performed as previously described (Banerji et al., 2016). Briefly, 0.1% tricaine solution was used for fish anaesthetization and their caudal fin rays amputated at 50% level using a sterile razor blade. Regenerating fins were harvested at the required time points and fixed in 4% paraformaldehyde (PFA) overnight at 4°C. The fixed fins were dehydrated in methanol (100%) and stored at 20°C until further use.

### MO-mediated gene knockdown in regenerating fins

The MOs used in the study were all fluorescein-tagged and purchased from Gene Tools, LLC. The sequences for MOs are as follows: (MO1) *smc3*-ATG blocking MO: 5′-TGTACATGGCGGTTTATGC-3′, (MO2) *smc3*-splice blocking MO: 5′-GCGTGAGTCGCATCTTACCTGTTTA-3′, *esco2* MO and Standard Control MO (Std-MO) from Banerji et al. (2016). MOs were reconstituted to a final concentration of 1 mM in sterile water. Microinjection and electroporation procedures were carried out as described in the previous studies (Banerji et al., 2016).

For synergy experiments between *esco2* or *smc3* and *cx43*, first the *esco2* and *smc3* MOs were tested at three different concentrations- 0.75 mM, 0.5 mM and 0.25 mM versus the Std control MO. No significant effect was observed in regenerate length and segment length for the 0.5 mM and 0.25 mM concentrations for both *esco2* MO and *smc3* MO1. Thus, the subthreshold concentration of 0.5 mM was selected for injecting and electroporating in 3 dpa *sof^b123^* heterozygote (*sof*/+) regenerating fins. Microinjection and electroporation procedures were carried out as described previously (Banerji et al., 2016).

### Measurements (regenerate length, segment length, cell proliferation and cell death)

MO-injected fins were calcein stained at 4 dpe/7 dpa, and regenerate length and segment length was determined as described (Du et al., 2001; Banerji et al., 2016). For detection of mitotic cells, H3P assay was performed on fins harvested at 1 dpe/4 dpa as described (Banerji et al., 2016). For detection of apoptotic cells, the TUNEL assay was performed as described in Banerji et al., 2016.

### RNA extraction and RT-PCR analysis on regenerating fins

RT-PCR analysis was performed on total mRNA extracted from 1 dpe/4 dpa harvested fins that were either injected with *smc3* splice blocking MO (MO2) or Std-MO injected. Trizol reagent (Gibco) was used to extract mRNA from a minimum of eight to 10 fins. For making cDNA, 1 mg of total RNA was reverse transcribed with SuperScript III reverse transcriptase (Invitrogen) using oligo (dT) primers. Two pairs of primers were used for testing the splicing efficiency. The control primer pair (C1-C2) was designed to amplify a portion of the exon 1 of *smc3* mRNA whereas the targeting primer pair (P1-P2) was designed to amplify the exon1 along with a portion of the intron1. The sequences of the control primers are as follows: C1 (forward primer) 5′-GACTGTTATGTCTTTTGCGTG-3′ and C2 (reverse primer) 5′ GCGGTTTATGCACAAAACACT-3′. The sequences of the targeting primers are as follows: P1 (forward primer) 5′-GGAGGAGGGTGTTTAATTCAGC-3′ and P2 (reverse Primer) 5′-GCTTCGAAAGCCTTGAATAATGAC-3′.

### qPCR analysis

qPCR analysis was performed on total mRNA extracted from 1 dpe/4 dpa harvested fins as described in the above section. The qPCR primers for *actin*, *cx43*, *hapln1a*, *sema3d*, *shh*, *spry4*, *mps1* were used at a concentration of 2.5 µM (Banerji et al., 2016; Govindan and Iovine, 2014, Table S1). Data from three biological replicates (3 dpa *esco2* MO, *smc3* MO2 and Std-MO injected fins) were used, with qPCR for each gene performed in duplicate as described in Banerji et al., 2016. *Actin* was used as a housekeeping gene and the delta C_T_ values represent expression levels normalized to *actin* values. Fold difference and standard deviation for the genes were determined using the method previously described (Sims et al., 2009; Ton and Kathryn Iovine, 2012; Banerji et al., 2016).

### RNA probe preparation for *in situ* hybridization on whole-mount and cryosectioned fin

The *cx43* template was made as described (Iovine et al., 2005). The *smc3* template was generated using gene-specific primers (Forward primer 5′-CAAACTGTGGTCGATCCCTTCAGC and reverse primer 5′-**TAATACGACTCACTATAGGG**GCTTCTCTTCAATCTTCT-3′). The RNA polymerase T7 (RT7) binding site is highlighted in bold for the reverse primer. Digoxigenin-labeled RNA probes were generated and whole mount/cryosection *in situ* hybridization was completed as previously described (Banerji et al., 2016).

### Transgenic overexpression of *cx43*

*Tg*(*hsp70:miR-133sp pd48*) denoted as transgene-positive (Tg+) and their siblings denoted as transgene-negative (Tg−) were used in the heat shock experiment as previously described (Banerji et al., 2016). Knocking down miR-133 (which targets *cx43* for degradation) via the ‘sponge’ transgene (three miR-133 binding sites) results in the increase of *cx43* levels (Yin et al., 2012).

### MO-mediated protein knockdown via electroporation in AB9 cells

AB.9 (ATCC^®^ CRL-2298™) is a primary fibroblast cell line originating from the zebrafish caudal fins. Once the cells were at 80-90% confluency in 100 mm dishes (28°C with 5% CO_2_) knockdown procedure was completed (Bhadra et al., 2015). Briefly, the adherent cells were washed with 1× PBS and trypsinized in 0.05% Trypsin-EDTA 1× (Gibco) for 5 min at 28°C. DMEM media supplemented with 15% heat inactivated FBS, antibiotics-antimycotics (Gibco) were added to inactivate the trypsin. The cells were collected by centrifugation at 750 rpm for 5 min. The pellet was re-suspended in 1-5 ml of HEPES buffer (115 mM NaCl, 1.2 mM CaCl_2_, 1.2 mM MgCl_2_, 2.4 mM K_2_PO_4_ and 20 mM HEPES with pH adjusted to 7.4) and put on ice. MOs were added to 400 µl of re-suspended cells in the cuvettes on ice and incubated for 5 min. The cells were electroporated at 170 V for 6-7 ms using an electroporater (Gene Pulser X Cell, BioRad). Electroporated cells were added to 1 ml of fresh media in 60 mm culture dishes and incubated at 28°C for 24 h.

### Lysate preparation and immunoblotting

Smc3 knockdown validation was confirmed by preparing MO1, MO2 and Std-MO injected fin lysates as described in Farwell et al. (2017). For evaluating the protein expression, western blotting technique using fluorescent secondary antibody was used as previously described (Farwell and Lowe-Krentz, 2016). AB9 cell lysate was prepared and western blots performed as previously described (Bhadra et al., 2015). The antibodies used for the western blots are as follows: Cx43, Esco2, Smc3, GFP and Tubulin were detected using anti-Cx43 (1:1000, Hoptak-Solga et al., 2008), anti-Esco2 (1:1000, Banerji et al., 2016), anti-Smc3 (1:1000, Santa Cruz Biotechnology, sc-8198), anti-GFP (1:1000, Clontech) and anti-α-Tubulin (1:1000, Sigma-Aldrich, T9026) respectively. The primary antibody step was followed by incubation in fluorophore-conjugated secondary antibodies for fin lysates. These include anti-rabbit Alexa Fluor 488 or 546 (1:10,000, Invitrogen), anti-mouse Alexa Fluor 488 or 546 (1:10,000, Invitrogen) and anti-goat Alexa Fluor 488 or 546 (1:10,000, Invitrogen). For western blots using heat-shocked fin lysates and cell lysates, the primary antibody step was followed by incubation in IgG-HRP (1:10,000, BioRad) secondary antibodies. The ECL chemiluminescent reagent (SuperSignal West Femto Maximum Sentivity Substrate, Pierce, Rockford, IL, USA) and X-ray films were used for signal detection. For measurement of band intensities and the percent change calculation, ImageJ software (https://imagej.nih.gov/ij/) was used. Relative pixel densities of gel bands were measured using the gel analysis tool in ImageJ software as previously described (Bhadra and Iovine, 2015). Tubulin was used as a loading control and thus the relative expression calculations were based on the ratio of Smc3 or Cx43 to Tubulin.

### Immunofluorescence on AB9 cells

Poly-L-lysine cover glasses were used for seeding the cells as previously described (Bhadra et al., 2015). Blocking was performed using 1% BSA for 1 h at room temperature. The cover slips were incubated with the primary antibody (see above) overnight at 4°C (in a covered chamber surrounded with damp Kim wipes). Cells were incubated with the secondary antibody for 1 h at room temperature (protected from light). The secondary antibodies used were as follows: anti-rabbit Alexa Fluor 488 or 568 (1:200, Invitrogen), anti-mouse Alexa Fluor 488 or 568 (1:200, Invitrogen). DAPI (1:1000, MP Biomedicals, LLC, Santa Ana, California, USA) labels the nucleus. Cells were mounted with Vectashield (Vector Laboratories) and examined with an Eclipse TE2000-U (Nikon, Melville, NY, USA) at 40× or 60×.

### ChIP

The ChIP protocol was performed on AB9 cells using a High-Sensitivity ChIP kit (Abcam, ab185913) according to the manufacturer's instruction. The procedure for monolayer or adherent cells was followed with few modifications. Briefly, cells were grown to 80–90% confluence on 100 mm dishes (around four to six dishes per round of ChIP), trypsinized and centrifuged at 1000 rpm for 20 min. The pellet was washed with 10 ml of 1× PBS and again centrifuged at the same speed and time. For cross-linking, 9 ml DMEM medium-containing formaldehyde (final concentration of 1%) was added to the cells and incubated at room temperature for 10 min on a rocker. After 10 min 1.25 M glycine solution was added and centrifuged at 1000 rpm for 20 min followed by a washing step with 10 ml of ice cold 1× PBS. After another round of centrifugation, lysis buffer with protease inhibitor was used to re-suspend the cell pellet (200 μl/1×106 cells) and incubated on ice for 30 min with periodic vortexing. The solution was centrifuged at 3000 rpm for 20 min and the chromatin pellet re-suspended with the ChIP buffer supplied in the kit (100 μl/1×106 cells). Chromatin was sheared using a tip sonicator (Branson sonfier cell disrupter 200, Thermo Fisher Scientific) with a 2.4 mm tip diameter microprobe (Qsonica P-3, Newtown, CT, USA) set to 25% power output. Sonication was carried out in three to four pulses of 10-15 s each, followed by 30-40 s rest on ice between each pulse. The sonicated chromatin was centrifuged at 12,000 rpm at 4°C for 10 min and stored at −20°C. A small amount of chromatin solution was used for DNA extraction in order to verify the size of the sheared DNA before starting the immunoprecipitation procedure (100-700 bp with a peak size of 300 bp). Antibody binding to assay wells and ChIP reactions was performed according to the manufacturer’s instructions. Antibodies used were anti-IgG (kit) and anti-Smc3 (Santa Cruz Biotechnology, sc-8198) with a concentration of 0.8 µg/well for both antibodies. The sealed strip wells with the respective antibodies and Antibody Buffer (kit) were incubated for 90 min at room temperature on an orbital shaker. The ChIP reaction was set up according to the low abundance target criteria (details provided in the protocol booklet) overnight at 4°C on an orbital shaker. The next day, the wells were washed with Wash buffer (kit) and DNA release buffer and cross-links were reversed (according to the manual). The released DNA was used in PCR or qPCR reactions.

### ChIP primer design and qPCR

The zebrafish *cx43* promoter sequence was obtained from the BAC clone (DKEY-261A18). Overlapping 31 primer pairs were designed spanning the entire 6.7 kb region of the *cx43* promoter (Table S2). For qPCR analysis, the primers were designed using the Primer Quest tool software (https://www.idtdna.com/Primerquest/Home/Index) from IDT (Table S3). Three independent samples (biological replicates) were prepared for ChIP, and qPCR reactions were performed in duplicate. ChIP DNA for non-immune IgG served as the negative control. The templates were a 1:10 dilution following ChIP using either IgG or Smc3 antibodies. PCR reactions were set up using SYBR green kit (Qiagen). Analyses of the amplified samples were performed using Rotor-Gene 6000 series software (https://www.qiagen.com/us/resources/resourcedetail?id=9d8bda8e-1fd7-4519-a1ff-b60bba526b57&lang=en) (Corbette Research) and the average cycle number (C_T_) determined for each amplicon. For fold enrichment calculation the *smc3* C_T_ values were normalized relative to IgG control values and were represented as delta C_T_ (ΔC_T_). The fold enrichment was determined using the ΔΔC_T_ method (2^−ΔΔ C^_T_) as described previously (Sims et al., 2009; Ton and Kathryn Iovine, 2012; Banerji et al., 2016). Statistical significance was determined by one-way ANOVA test (*P*<0.001) with Tukey's multiple comparison post hoc test (using MINITAB 17 software.)

### Statistical analysis

All graphs and error bars were generated using Microsoft Excel (2013) software. For statistical significance calculation, two-tailed unpaired *t*-test was performed using Graphpad software (www.graphpad.com). Statistical significance was also determined by one-way ANOVA (*P*<0.001) with Tukey's multiple comparison post hoc test (using MINITAB 17 software).

## Supplementary Material

Supplementary information

First Person interview
