# Supplementary material for: Cohesin mediates Esco2-dependent transcriptional regulation in a zebrafish regenerating fin model of Roberts Syndrome
Source: Biol Open. 2017 Oct 30;6(12):1802–13. doi: 10.1242/bio.026013 (PMC5769645; doi:10.1242/bio.026013)
Supplement: Supplementary information [file biolopen-6-026013-s1.pdf]

© 2017. Published by The Company of Biologists Ltd | Biology Open (2017): doi:10.1242/bio.026013: Supplementary information

FIRST PERSON

First person – Rajeswari Banerji

First Person is a series of interviews with the first authors of a selection of papers published in Biology Open, helping early-career researchers promote themselves alongside their papers. Rajeswari Banerji is first author on 'Cohesin mediates Esco2-dependent transcriptional regulation in zebrafish regenerating fin model of Roberts Syndrome', published in BiO. Rajeswari conducted the research in this article while a PhD student in the lab of M. Kathryn Iovine and Robert Skibbens at Lehigh University, PA, USA. She is currently a postdoctoral fellow in Manisha Patel's lab at the Skaggs School of Pharmacy, University of Colorado, investigating learning mechanisms of paediatric diseases in order to find pharmacological targets for proper treatment.

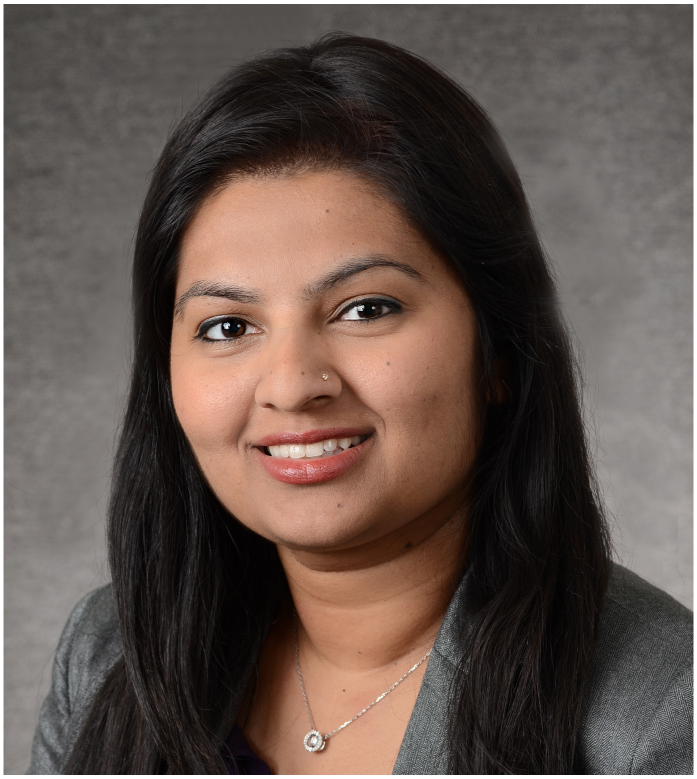

Rajeswari Banerji

What is your scientific background and the general focus of your lab?

I recently completed my PhD in the Department of Cell and Molecular biology under the mentorships of Kathryn Iovine and Robert Skibbens. The Skibbens lab studies the role of cohesins and their associated factors using yeast as a model system. These factors such as ESCO2 are clinically significant as defects lead to various developmental disorders such as Robert's syndrome (RBS). The Iovine lab examines the role of the gap junction protein Cx43, which is also critical during skeletal regeneration. For instance, Cx43 mutations in humans results in oculodentodigital dysplasia (ODDD). My PhD research work was based on a collaboration between the two labs to develop a vertebrate model system to understand RBS and the mechanistic overlap with ODDD, using the zebrafish regenerating caudal fin as the model system.

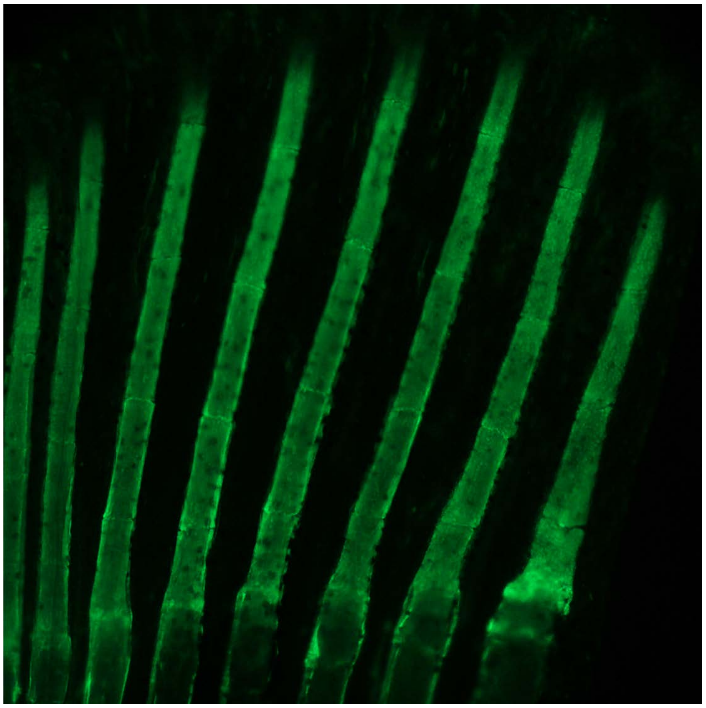

Zebrafish caudal fin stained with calcein

Rajeswari Banerji's contact details: Skaggs School of Pharmacy, University of Colorado, Anschutz Medical Campus, Aurora, CO 80045, USA.

E-mail: rajeswari.banerji@ucdenver.edu

What are the potential implications of these results for your field of research?

Our research identifies a novel transcription-based mechanism underlying RBS which is similar to CdLS. Thus, our study suggests that the underlying causation for both diseases are related – a link largely undeveloped in the field. The unified mechanism provides opportunities to find a single drug target from which new pharmaceutical treatments can be developed in the future.

How would you explain the main findings of your paper to non-scientific family and friends?

RBS is a severe type of human developmental disorder characterized by craniofacial deformities, limb malformation and mental retardation. Currently the treatment options are very limited and not very beneficial. Thus, it is important to understand the molecular mechanisms underlying RBS if we are to identify relevant pharmacological targets for more effective treatments. In a previous study we developed a zebrafish regenerating fin vertebrate model to

examine the skeletal defects of RBS. We established a genetic link between the gene mutated in RBS – *esco2* – and the clinically relevant gap junction gene *cx43*, which suggested a transcriptional role for Esco2 in Cx43 regulation. This is the first study to provide evidence that unifies RBS and similar birth defect maladies such as Cornelia de Lange syndrome (CdLS).

What, in your opinion, are some of the greatest achievements in your field and how has this influenced your research?

One of the greatest achievements in our field is the use of various model systems such as zebrafish, *Drosophila* and yeast to study mechanisms underlying human diseases. In particular zebrafish research plays a valuable role in developmental studies and also drug discovery.

What has surprised you the most while conducting your research?

Both RBS and CdLS are severe birth defects with overlapping phenotypes, and arise from mutation of genes that function in a common pathway, but surprisingly the etiologies of these syndromes are believed to be distinct. Though it is widely accepted that CdLS arises from transcriptional deregulation of a set of genes, the mechanism underlying RBS remains unknown. A popular model states that RBS is caused by mitotic failure and cell death that is completely distinct from CdLS. I was surprised that similar developmental diseases such as RBS and CdLS could have such distinct mechanisms and believed that this area needed further investigation.

What changes do you think could improve the professional lives of early-career scientists?

I feel collaboration plays a beneficial role in any kind of research. I was fortunate to work in such an environment that gave me different perspectives and ideas that helped me progress in my work and shape my future. I highly recommend the kind of exposure one receives by pursuing collaborative research.

"I highly recommend the kind of exposure one receives by pursuing collaborative research."

What's next for you?

Currently I am working as a postdoctoral researcher in the Department of Pharmacy. I strongly believe that zebrafish is an ideal model for paediatric research and high-throughput drug testing. At present I am working on a severe form of childhood epilepsy using zebrafish larvae and testing various drugs to decrease the severe seizures associated with this disease. In the long run I would like to continue with drug discovery and ultimately enter in the area of clinical trial studies.

Reference

Banerji, R., Skibbens, R. V. and Iovine, M. K. (2017). Cohesin mediates Esco2-dependent transcriptional regulation in zebrafish regenerating fin model of Roberts Syndrome. *Biol. Open* **6**, doi:10.1242/bio.026013.
